# Characterization of the Response of Primary Cells Relevant to Dialysis-Related Amyloidosis to β_2_-Microglobulin Monomer and Fibrils

**DOI:** 10.1371/journal.pone.0027353

**Published:** 2011-11-09

**Authors:** Morwenna Y. Porter, Katy E. Routledge, Sheena E. Radford, Eric W. Hewitt

**Affiliations:** Astbury Centre for Structural Molecular Biology and Institute of Molecular and Cellular Biology, University of Leeds, Leeds, United Kingdom; University of Canterbury, New Zealand

## Abstract

The formation of insoluble amyloid fibrils is associated with an array of devastating human diseases. Dialysis-related amyloidosis (DRA) is a severe complication of hemodialysis that results in the progressive destruction of the bones and joints. Elevated concentrations of β_2_-microglobulin (β_2_m) in the serum of subjects on hemodialysis promote the formation of amyloid fibrils in the osteoarticular tissues, but the cellular basis for the destruction of these tissues in DRA is poorly understood. In this study we performed a systematic analysis of the interaction of monomeric and fibrillar β_2_m with primary human cells of the types present in the synovial joints of subjects with DRA. Building upon observations that macrophages infiltrate β_2_m amyloid deposits *in vivo* we demonstrate that monocytes, the precursors of macrophages, cannot degrade β_2_m fibrils, and that both monomeric β_2_m and fibrillar β_2_m are cytotoxic to these cells. β_2_m fibrils also impair the formation of bone resorbing osteoclasts from monocytes and reduce the viability of osteoblasts, the cell type that produces bone. As a consequence, we predict that β_2_m amyloid will disrupt the remodelling of the bone, which is critical for the maintenance of this tissue. Moreover, we show that β_2_m fibrils reduce the viability of chondrocytes, rationalizing the loss of cartilage in DRA. Together, our observations demonstrate that β_2_m cytotoxicity has multiple cellular targets in the osteoarticular tissues and is likely to be a key factor in the bone and joint destruction characteristic of DRA.

## Introduction

The formation of insoluble amyloid fibrils is associated with a spectrum of devastating human diseases, many of which are characterised by tissue destruction [1]. One such disorder is dialysis-related amyloidosis (DRA), a debilitating complication of long-term hemodialysis [2], [3]. The culprit protein of DRA is β_2_-microglubulin (β_2_m) [4], [5], the non-covalent light-chain of cell surface major histocompatibility complex (MHC) class I molecules [6]. Upon dissociation from MHC molecules, β_2_m is normally removed from the bloodstream by the kidneys [6]. The normal serum concentration of β_2_m is 1–3 µg/ml but, in end stage renal disease, neither the kidney nor the dialysis membrane can efficiently remove β_2_m from the circulation and serum levels increase by up to 60 fold and can exceed 100 µg/ml [2], [3], [7]. At these elevated concentrations, β_2_m forms amyloid fibrils in the osteoarticular tissues resulting in arthropathy, cartilage destruction, bone cysts leading to pathologic fractures, carpal tunnel syndrome and spondyloarthropathy [2]–[6], [8], [9].

β_2_m fibril formation is promoted by collagen and the glycosaminoglycans (GAGs) chondroitin-sulfate and heparin [10]–[14]. Collagen and chondroitin-sulfate are abundant in osteoarticular tissues, rationalizing the deposition of β_2_m amyloid at these sites, whereas heparin is an anti-coagulant used in hemodialysis. β_2_m lacking the N-terminal six residues of the mature protein (ΔN6β_2_m) constitutes ≤30% of the β_2_m in *ex vivo* DRA amyloid [15]. Unlike full-length wild type (WT) β_2_m, ΔN6β_2_m forms amyloid fibrils *de novo* at neutral pH *in vitro*, a process that is enhanced by GAGs [12], [13], [16]–[18]. ΔN6β_2_m fibrils also cross-seed fibril formation with WT β_2_m and can convert WT β_2_m into an amyloidogenic state, suggesting that ΔN6β_2_m could initiate fibril formation *in vivo* [17], [18].

In contrast to the increasing knowledge of the mechanism of β_2_m fibril assembly *in vitro* [6], [19], how β_2_m amyloid causes skeletal morbidity in DRA is poorly understood. Macrophages infiltrate β_2_m amyloid deposits and have been implicated in the development of symptomatic DRA [20]–[22]. Cells of the monocyte/macrophage lineage are precursors of osteoclasts, which may be responsible for osteolytic lesions in DRA [23]–[27]. Indeed, human β_2_m has been shown to promote osteoclastogenesis from murine macrophages [28], but whether monomeric or fibrillar β_2_m species promote human osteoclast formation is not known. Since β_2_m fibrils generated *in vitro* are cytotoxic to some cultured cell lines [29], β_2_m amyloid formation could also cause bone and joint destruction via cytotoxicity to cell types that are responsible for the maintenance of the osteoarticular tissues.

Herein we perform a systematic comparison of the effects of monomeric and fibrillar β_2_m on primary human cells relevant to the pathology of DRA (monocytes, osteoblasts and chondrocytes). Our data do not support a role for monomeric or fibrillar β_2_m in osteoclast formation by human monocytes, but instead show that β_2_m monomer and fibril preparations are cytotoxic to monocytes, osteoblasts and chondrocytes and hence β_2_m cytotoxicity may be an important factor in the bone and joint destruction associated with DRA.

## Results

### Generation of β_2_m fibrils in vitro at a physiologically relevant pH

**Figure 1:**
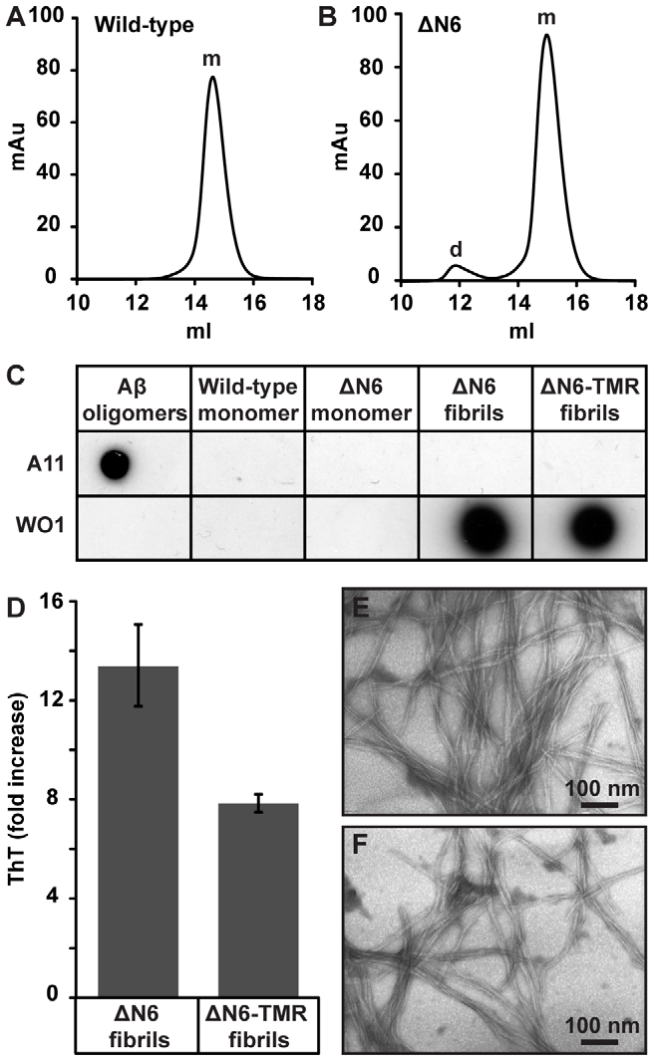
Analysis of monomeric and fibrillar β_2_-microglobulin (β_2_m). Analytical size exclusion chromatography traces of 1 mg/ml full-length wild type (WT) β_2_m (a) and ΔN6β_2_m (ΔN6) (b), obtained in phosphate buffered saline pH 7.3 at 25°C. Monomer (m) and dimer (d) peaks are indicated. (c) Immunoblots of monomeric and fibrillar β_2_m probed with A11 anti-oligomer and WO1 anti-fibrillar antibodies. Aβ_1–40_ oligomers were used as a positive control for the A11 antibody. (d) Thioflavin-T fluorescence of unlabelled and TMR-labelled β_2_m fibrils, error bars represent one standard deviation over 60 readings. Negative stain transmission electron microscopy images of unlabelled (e) and TMR labelled (f) β_2_m fibrils.

Recombinant human WT β_2_m and ΔN6β_2_m were expressed in *E. coli* and purified [12], [30], [31]. Size-exclusion chromatography (SEC) confirmed that both proteins are predominantly monomeric at pH 7.3 (≥98% of WT β_2_m; ∼96% of ΔN6β_2_m), although ∼4% of ΔN6β_2_m was dimeric (Fig. 1A and B). Neither protein was recognized by the A11 or WO1 antibodies (Fig. 1C), which bind generic epitopes in cytotoxic oligomers and amyloid fibrils, respectively [32], [33].

To generate β_2_m fibrils at a physiologically relevant pH, and within an experimentally tractable timescale, fibrils generated at pH 2 from WT β_2_m were fragmented to produce seeds that were then extended with ΔN6β_2_m monomer at pH 7.3 in the presence of heparin [12]. The resultant fibrils were not recognized by the A11 antibody, were recognized by the WO1 antibody (Fig. 1C); bound the amyloid specific dye thioflavin-T (Fig. 1D) and exhibited a long straight morphology when visualized by negative stain transmission electron microscopy (TEM) (Fig. 1E) reminiscent of *ex vivo* β_2_m amyloid [34]–[36]. Moreover, we have shown previously with Fourier transform infrared spectroscopy that fibrils elongated from WT β_2_m seeds at pH 7 closely resemble *ex vivo* β_2_m amyloid [36]. Fibrils formed from seeds elongated with a 1∶9 mixture of ΔN6β_2_m and 5-(and -6)-carboxytetramethylrhodamine-SE (TMR) labelled ΔN6β_2_m also had the characteristic features of amyloid (Fig. 1C, D and F).

### Human monocytes internalize, but do not degrade, β_2_m fibrils

**Figure 2:**
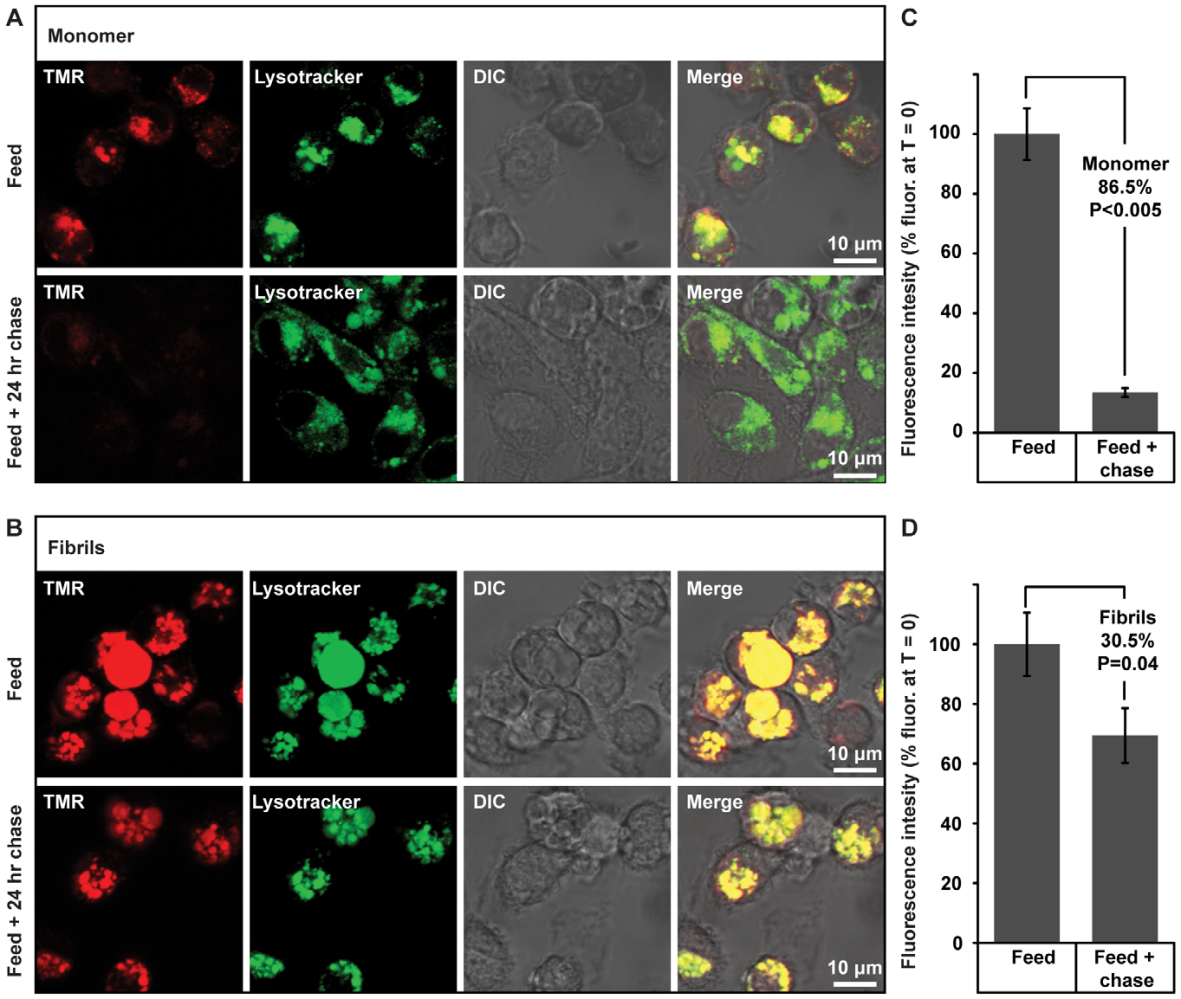
Internalization and degradation of monomeric and fibrillar β_2_-microglobulin (β_2_m) by primary human monocytes. Monocytes were incubated with 10 µg/ml of ΔN6β_2_m monomer (90% unlabelled ΔN6β_2_m, 10% TMR-labelled ΔN6β_2_m) (a) or TMR-labelled fibrils formed from a 9∶1 mixture of unlabelled: labelled ΔN6β_2_m (red, feed) for 24 hours (b). Lysosomes were visualized by staining with LysoTracker green prior to confocal microscopy. Differential interferance contrast (DIC) images are also shown. Cells were then washed to remove non-cell associated protein and chased for a further 24 hours before imaging again (feed +24 hour chase). The mean intracellular fluorescence of 20 cells was quantified for cells incubated with monomeric β_2_m (c) and fibrillar β_2_m (d) before and after the 24 hour chase. Values are normalized to 100% for the intracellular fluoresence of cells before the chase and the percentage reduction after 24 hour chase is indicated. Error bars represent the SEM; P values differing from the PBS control were calculated by the Student's t-test and are stated.

The macrophages that infiltrate β_2_m amyloid plaques are present around blood vessels consistent with these cells being derived from peripheral blood monocytes [20], [37]. We, therefore, investigated whether these cells could play a protective role in DRA via the degradation of β_2_m fibrils. CD14^+^ monocytes isolated from peripheral blood were cultured in the presence of macrophage colony stimulating factor (M-CSF), whereupon they become adherent and develop a macrophage-like morphology. These cells were incubated with either TMR-labelled monomeric ΔN6β_2_m or TMR-labelled β_2_m fibrils. Prior to imaging by confocal microscopy the cells were incubated with LysoTracker green, a fluorescent probe that accumulates in acidic intracellular compartments. After 24 hours, both monomer and fibrils were internalized and co-localized with Lysotracker green in punctate structures (Fig. 2A and B). The smaller puncta stained by Lysotracker green are characteristic of lysosomes, whereas the larger punctate structures that are most prominent in cells incubated with fibrils may correspond to acidified phagosomes. Indeed, this would be consistent with electron microscopy studies of *ex vivo* DRA amyloid, in which macrophages were shown to phagocytose β_2_m amyloid [38].

After incubation for a further 24 hours in the absence of extracellular TMR-labelled monomeric ΔN6β_2_m or fibrils, a pronounced decrease in intracellular fluorescence was observed for cells that had internalized monomeric protein (Fig. 2A and C). In contrast only a modest reduction in intracellular fluorescence occurred in cells that had internalized β_2_m fibrils (Fig. 2B and D). Thus monocytes internalize and traffic both β_2_m monomer and fibrils to acidic vesicles, but only the monomeric protein is degraded efficiently. The inability of these phagocytic cells to degrade β_2_m fibrils would allow amyloid deposition to proceed unchecked in the synovial joints of subjects with DRA.

### Monomeric and fibrillar β_2_m do not promote osteoclast formation

In addition to forming macrophages, monocytes can differentiate into bone resorbing osteoclasts [24]–[26]. Since primary human monocytes interact with both monomeric and fibrillar β_2_m (Fig. 2), and human β_2_m monomer has been shown to stimulate osteoclast formation from murine macrophages [28], we next examined whether either monomeric or fibrillar β_2_m can promote osteoclast formation from human monocytes. Osteoclasts are defined as large multinucleate cells, which express high levels of tartrate-resistant acid phosphatase (TRAP) and can resorb artificial bone substrates [24]–[26]; hence each of these characteristics were used to monitor osteoclast formation.

**Figure 3:**
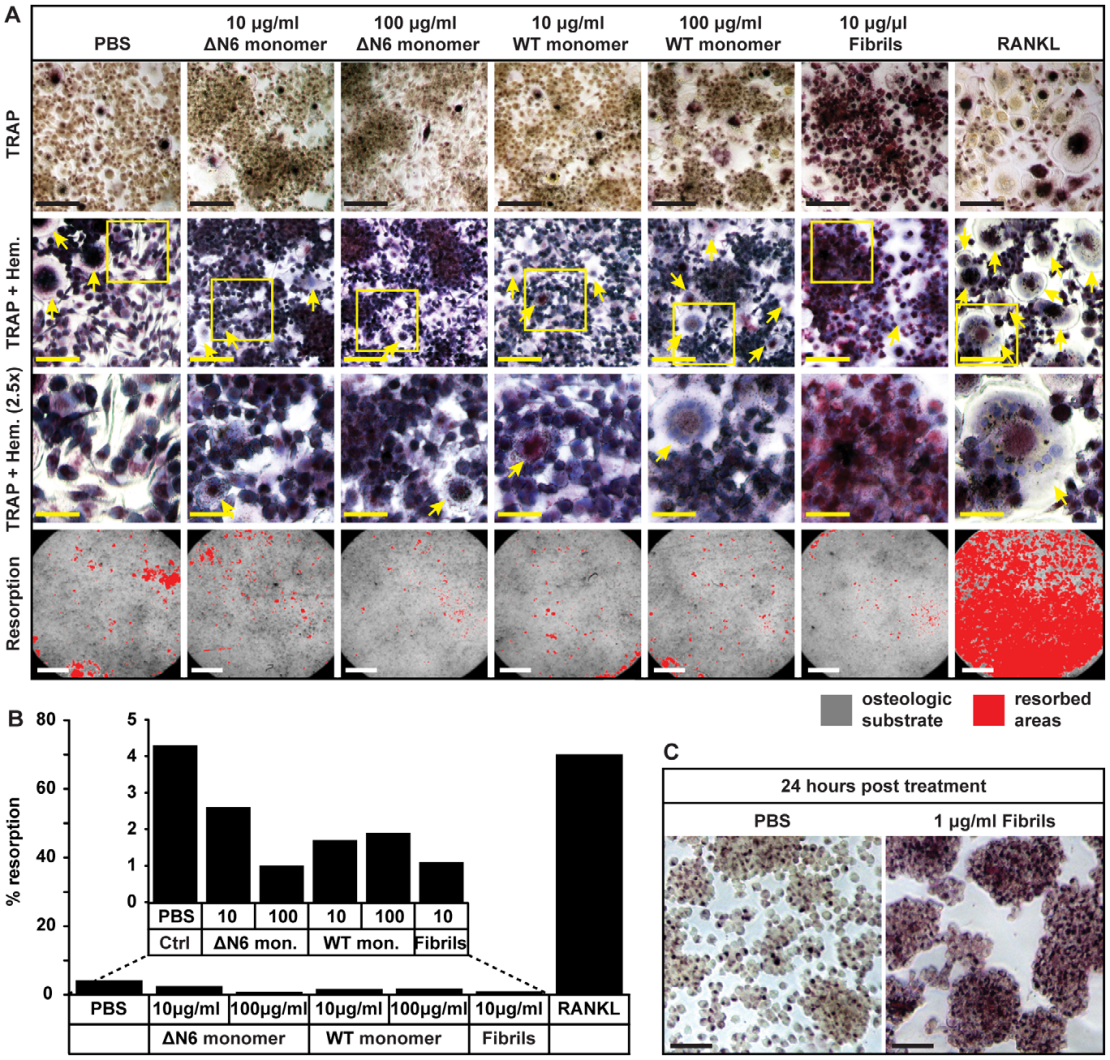
Effects of monomeric or fibrillar β_2_-microglobulin (β_2_m) on primary human monocyte differentiation into osteoclasts. Monocytes were incubated with the indicated substances and macrophage colony-stimulating factor (M-CSF) for 14 days. RANKL induces osteoclast formation and was used as a positive control. (a) Top row (scale bar 50 µm): cells stained for the osteoclast marker, TRAP. 2nd row (scale bar 50 µm): TRAP stained cells were counter stained with hematoxylin to visualize cell morphology (nuclei, blue), arrows indicate large multinucleate cells. Regions within the yellow boxes are magnified in the 3rd row (scale bar 20 µm). Bottom row (scale bar 1 mm): resorption of an osteologic substrate by the cells treated as indicated. Resorbed areas are coloured red. (b) Resorption was quantified and results from a representative experiment are shown. The inset shows an expansion of the first six columns. (c) Monocytes were incubated with either PBS control or β_2_m fibrils for 24 hours, and then stained for TRAP (scale bar 50 µm).

In a control experiment, monocytes were incubated with receptor activator of nuclear factor-Κ ligand (RANKL), a cytokine that promotes osteoclast formation [24]–[26]. After 14 days there were abundant large (≥20 µm) cells, which stained positive (dark pink) for TRAP in centrally located intracellular vesicles (Fig. 3A). Many of the cells were multinucleate, as visualised by hematoxylin staining, and they were able to resorb the majority (∼70%) of an artificial bone substrate (Fig. 3A and B). This confirmed that under the culture conditions used in this study osteoclasts form in 14 days. In contrast, when cells were incubated with buffer, ΔN6β_2_m monomer or WT β_2_m monomer, at concentrations (10 and 100 µg/ml) that span those observed in the serum of subjects with DRA [2], [3], [7], only a small number of multinucleate, TRAP positive cells were formed, and little of the artificial bone substrate was resorbed (<5%) (Fig. 3A and B) indicating that neither WT β_2_m nor ΔN6β_2_m monomeric proteins promote the differentiation of human monocytes into osteoclasts.

When monocytes were incubated with 10 µg/ml β_2_m fibrils very few large multinucleate cells were formed and there was limited resorption of the artificial bone substrate (Fig. 3A and B), thus demonstrating that β_2_m fibrils do not promote osteoclast formation. Interestingly many cells did, however exhibit a dramatic increase in TRAP positive vesicles, giving them a dark pink appearance after staining for the enzyme (Fig. 3A). Elevated TRAP expression has also been observed in pulmonary alveolar macrophages that have phagocytosed microbes and other particles [39]; therefore TRAP could be increased in monocytes due to the internalization of β_2_m fibrils. Indeed after only 24 hours incubation with β_2_m fibrils, the level of TRAP expression was increased markedly (Fig. 3C).

**Figure 4:**
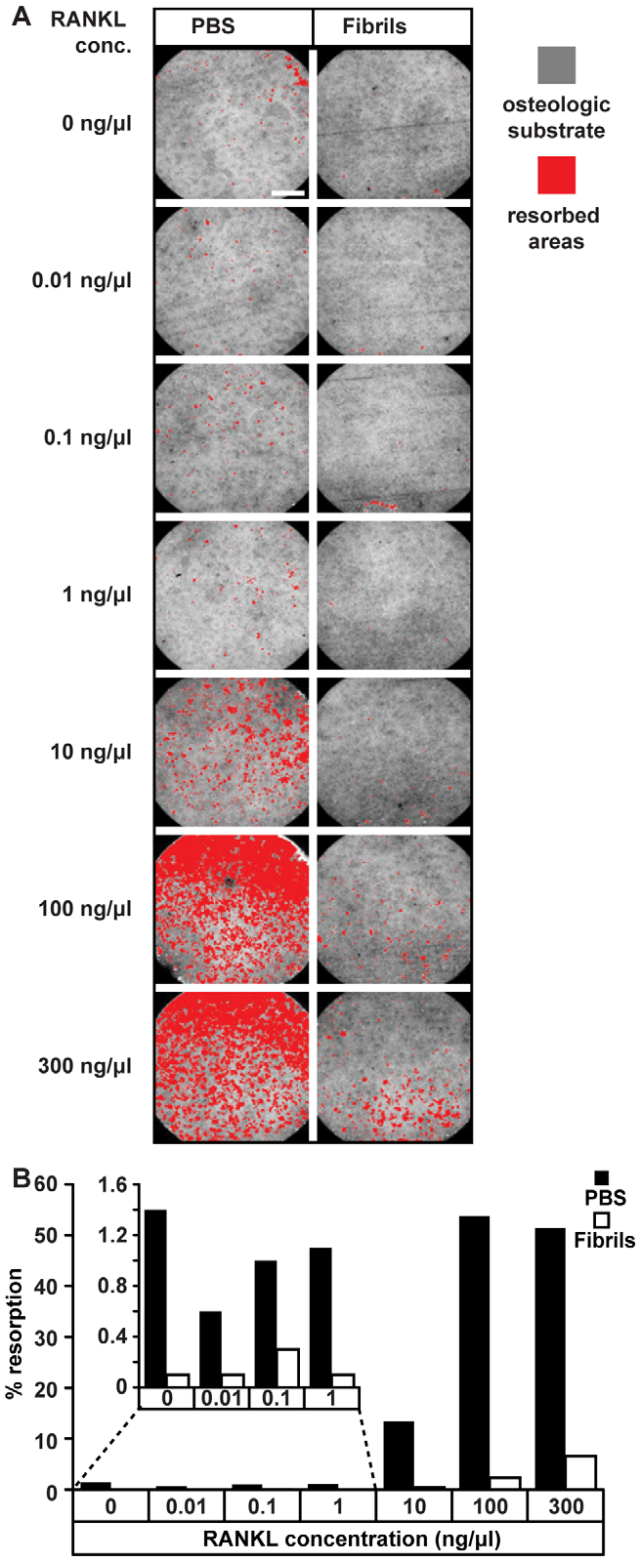
Effects of β_2_-microglobulin (β_2_m) fibrils on RANKL-dependent osteoclast formation. Primary human monocytes were incubated with M-CSF and the indicated concentrations of RANKL in the presence of PBS or 10 µg/ml β_2_m-fibrils for 14 days and their ability to resorb an osteologic substrate was then assessed. (a) Images of the osteologic substrate following the removal of cells. Resorbed regions are highlighted in red, scale bar 1 mm. (b) Resorption was quantified and results from a representative experiment are shown. The inset shows an expansion of the first four columns.

An alternative possibility for the observed increase in TRAP expression was that β_2_m fibrils may provide a partial osteoclastogenic signal that synergizes with other signalling molecules to promote the formation of mature osteoclasts. *In vivo* osteoclastogenesis is promoted by RANKL, a cytokine expressed on the surface of osteoblasts [24]–[26], this molecule was absent from all but the positive control cultures. We, therefore, examined whether β_2_m fibrils act in synergy with RANKL to promote osteoclast formation. Monocytes were incubated with increasing concentrations of RANKL in either the presence or absence of 10 µg/ml β_2_m fibrils and osteoclast function assessed by measuring resoprtion of the artificial bone substrate. As expected when monocytes were incubated with RANKL alone the number of osteoclasts formed and resorption of the artificial bone substrate increased as the concentration of this cytokine was increased (Fig. 4A and B). However, when cells were incubated with RANKL and β_2_m fibrils, a reduction in resorption of the artificial bone substrate was observed relative to cells incubated with RANKL alone (Fig. 4A and B). Thus although β_2_m fibrils increase TRAP expression they do not promote osteoclast formation, instead β_2_m fibrils reduce RANKL-dependent osteoclast formation.

### β_2_m fibrils are cytotoxic to monocytes, osteoblasts and chondrocytes

**Figure 5:**
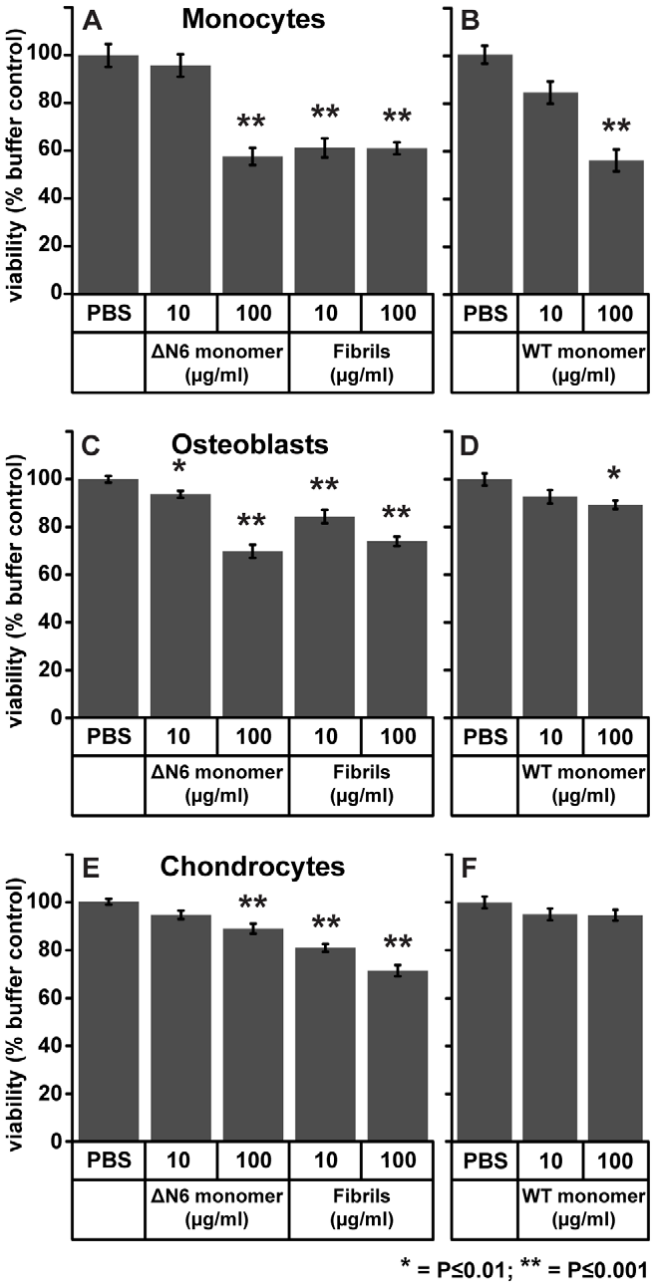
Effects of monomeric and fibrillar β_2_-microglobulin (β_2_m) on primary human monocytes, osteoblasts and chondrocyte viability. Cells were incubated with the indicated substances for 24 hours and then their ability to reduce MTT, as a measure of viability, was measured. (a) Effects of β_2_m fibrils and ΔN6β_2_m (ΔN6) monomer on monocyte viability; the mean from three donors is plotted. (b) Effects of full-length wild type (WT) β_2_m monomer on monocyte viability; the mean from three donors is plotted. (c) The effects of β_2_m fibrils and ΔN6β_2_m monomer on osteoblast viability; the mean from four donors is plotted. (d) The effect of WT β_2_m monomer on osteoblast viability; the mean from one donor is plotted. (e) The effect of β_2_m fibrils and ΔN6β_2_m monomer on chondrocyte viability; the mean from four donors is plotted. (f) Effects of WT β_2_m monomer on chondrocyte viability, the mean from one donor is plotted. The data from individual donors for monocytes, osteoblasts and chondrocytes are presented in Figures S1, S2B and S3B, respectively. Error bars represent the SEM; P values differing from the PBS control were calculated by the Student's t-test, * = P≤0.01; ** = P≤0.001.

Previously we have shown that β_2_m fibrils are cytotoxic to the murine macrophage cell line RAW 264.7 [29]. We therefore investigated whether β_2_m fibrils are also cytotoxic to primary human monocytes. Using the 3-(4,5-dimethyl-2-thiazolyl)-2,5-diphenyl-2H-tetrazolium bromide (MTT) assay, a pronounced and significant reduction in cell viability was observed for monocytes incubated with 10 µg/ml β_2_m fibrils (Fig. 5A and S1A). By contrast to the fibrils, 10 µg/ml of ΔN6β_2_m and WT β_2_m had a minimal effect on the viability of monocytes (Fig. 5A, 5B, S1A and S1B). Thus fibrils are more cytotoxic than monomeric β_2_m, which is consistent with our previous study of the effect of monomeric and fibrillar β_2_m on the viability of cell lines [29]. β_2_m fibril cytotoxicity may contribute to the failure of monocytes to degrade β_2_m fibrils and could also be responsible for the observed reduction in ostoeclast formation, as monocyte death would prevent these cells from fusing together to form osteoclasts. However, at 100 µg/ml, β_2_m fibrils reduced cell viability by ∼40%, as did the monomeric forms of ΔN6β_2_m and WT β_2_m (Fig. 5A and S1A), demonstrating that monomeric forms of β_2_m are also cytotoxic at this concentration.

Next we investigated whether β_2_m could contribute to bone and joint destruction in DRA by reducing the viability of osteoblasts and chondrocytes, the cells that produce bone and cartilage respectively. The association of TMR-labelled ΔN6β_2_m monomer or β_2_m fibrils with primary human osteoblasts and chondrocytes was visualized by confocal microscopy. After 24 hours, monomeric TMR-labelled ΔN6β_2_m was present in punctate intracellular structures in osteoblasts and chondrocytes, consistent with internalisation of the protein (Fig. S2A and S3A). Similar internalization was seen when β_2_m fibrils were incubated with chondrocytes, while fibrils added to osteoblasts remained in close proximity to the cell surface as large aggregates, with some limited cell-associated punctate fluorescence indicative of cell association and internalization (Fig. S2A and S3A). Our observations suggest both cell types internalize monomeric β_2_m, although chondrocytes internalize β_2_m-fibrils more readily than osteoblasts.

The effect of β_2_m monomer and fibrils on osteoblast and chondrocyte viability was then assessed; both 10 and 100 µg/ml β_2_m fibrils caused a significant reduction in the viability of osteoblasts and chondrocytes (Fig. 5C, 5E, S2B and S3B). ΔN6β_2_m monomer also reduced cell viability of both cell types (Fig. 5C, 5E, S2B and S3B). In contrast to monomeric ΔN6β_2_m, 100 µg/ml of WT β_2_m monomer had no significant effect on chondrocyte viability and caused only a limited decrease in viability of osteoblasts, at the highest concentration used (Fig. 5D and F). Taken together, these data suggest that monomeric ΔN6β_2_m and fibrillar β_2_m cytotoxicity to chondrocytes and osteoblasts are important factors in the pathology of DRA. Chondrocyte cell death would be predicted to decrease formation of new cartilage, while the reduction in osteoclast formation in combination with the death of osteoblasts may impair bone turnover in subjects with DRA.

## Discussion

**Figure 6:**
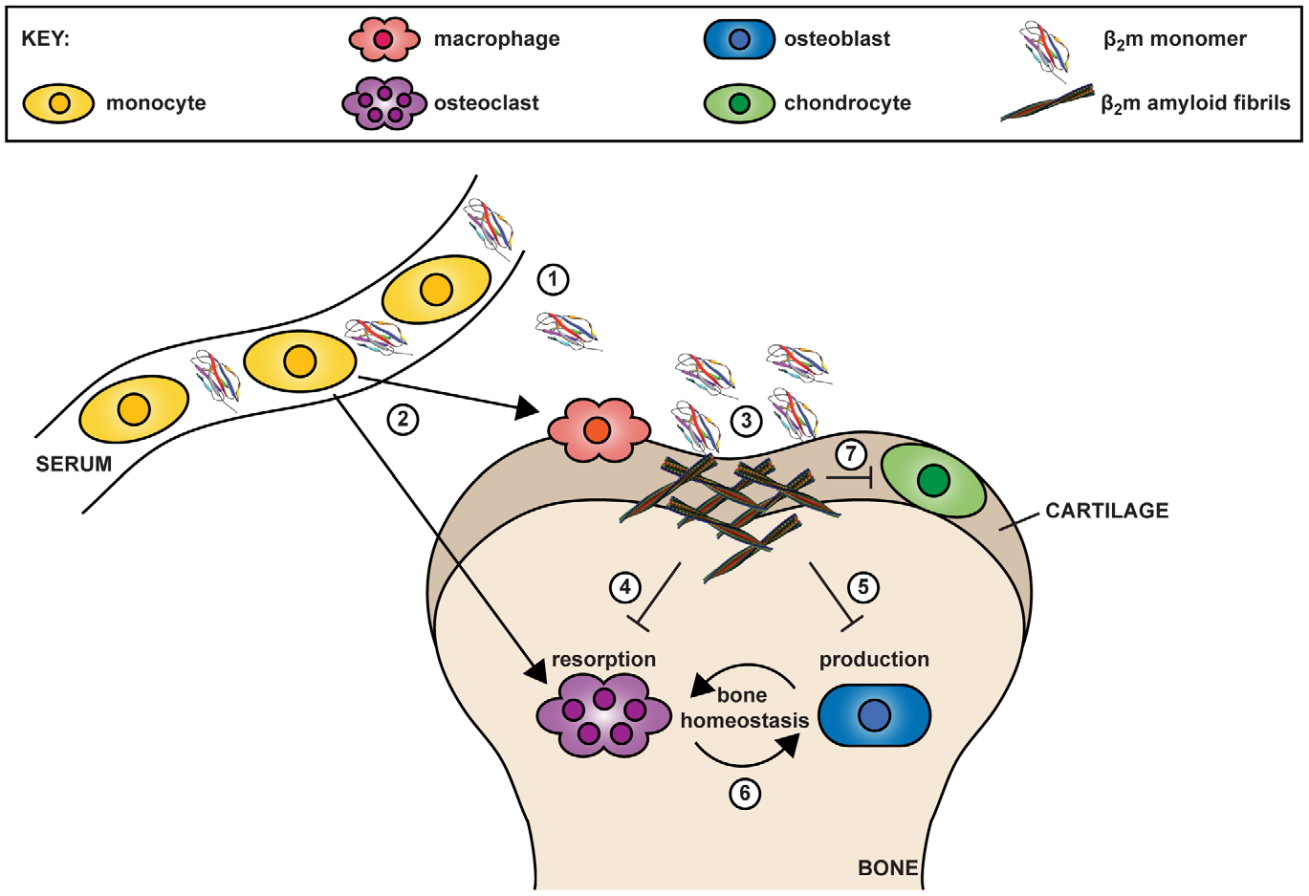
Model for a role of monomeric and fibrillar β_2_-microglobulin (β_2_m) cytotoxicity in dialysis related amyloidosis. (1) Elevated levels of circulating β_2_m monomer form amyloid deposits in osteoarticular tissues. (2) Circulating monocytes are able to differentiate into both macrophages and osteoclasts. (3) Monocyte-derived macrophages infiltrate β_2_m amyloid deposits, but the resistance of the amyloid fold to proteolysis in combination with β_2_m amyloid cytotoxicity, prevents the clearance of the amyloid deposits. (4) β_2_m amyloid induced monocyte/macrophage cell death also contributes to reduced osteoclast formation, causing a decrease in the resorption of old bone. (5) Osteoblast cell death in the presence of β_2_m monomer and/or fibrils causes decreased bone formation. (6) Decreased bone resorption and formation contribute to pathological alterations to normal bone homeostasis. (7) Chondrocyte cell death in the presence of β_2_m monomer and/or fibrils causes decreased cartilage formation, which could allow gradual loss of this tissue and exposure of bone to cytotoxic β_2_m monomer and fibrils.

Despite the identification of β_2_m as the culprit protein of DRA in 1985 [4], [5], how β_2_m amyloid causes bone and joint destruction in DRA remains poorly understood. In order to elucidate the cellular basis for the pathological consequences in DRA we performed a systematic analysis of the effects of β_2_m fibrils and monomeric β_2_m on cell types that are present in the synovial joints of subjects with DRA. Our data suggest that the bone and joint destruction characteristic of DRA is a multifactorial process in which β_2_m amyloid fibrils and elevated concentrations of monomeric β_2_m are cytotoxic to multiple cellular targets (Fig. 6).

We demonstrate that primary human monocytes internalize, but do not degrade, β_2_m fibrils. This is consistent with the resistance of the β_2_m amyloid fold to lysosomal proteolysis [40], [41], but could be exacerbated by the cytotoxicity of β_2_m fibrils to the monocytic precursors of macrophages. The death of macrophages that infiltrate amyloid deposits *in vivo* [20]–[22], [37], would be predicted to prevent the clearance of β_2_m monomer and fibrils allowing the continued accumulation of β_2_m amyloid in the synovial joints.

Cells of the monocytic lineage are also the precursors of osteoclasts, which in combination with osteoblasts are responsible for bone remodelling [26]. Monomeric β_2_m did not promote osteoclast formation from human monocytes. This contrasts with a recent study in which monomeric β_2_m was shown to promote osteoclast formation from murine macrophages [28], and implies that β_2_m has differential effects on human and mouse cells. Similarly, β_2_m fibrils did not promote the formation of bone resorbing osteoclasts despite increased expression of TRAP, as there was no increase in the number of multinucleate cells nor did β_2_m fibrils promote the resorption of the artificial bone substrate. Once considered just as a marker of osteoclasts, it has become clear that TRAP has a number of important roles in both the skeleton and the immune system, and is expressed by a number of different cell types including macrophages [39], [42]. Thus the increase in TRAP expression in monocytes may be the result of the activation of these cells by β_2_m fibrils. Moreover, we found that β_2_m fibrils impaired RANKL-induced osteoclast formation. This was presumably as a consequence of cytotoxicity to monocytes, as fibril-induced cell death of the monocytic precursors would be predicted to prevent these cells from fusing together to form multinucleate osteoclasts. This reduction in osteoclast formation, in combination with the reduction in viability of osteoblasts caused by β_2_m fibrils and to a lesser extent monomeric β_2_m, suggests that bone remodelling will be perturbed in DRA. The formation of bone cysts in DRA may, therefore, be the consequence of a reduction in the turnover of damaged bone, rather than an increase in osteoclast activity. Moreover, the effect of β_2_m fibrils on osteoclast formation and the viability of osteoblasts may exacerbate the reduction in bone turnover that is a common feature of renal osteodystrophy associated with chronic kidney disease [43]. β_2_m fibrils were also shown to be cytotoxic to chondrocytes, rationalizing why the formation of β_2_m amyloid deposits in cartilage results in the loss of this tissue [2], [3], [8], [9], [22], which could in turn expose the bone surface to the cytotoxic effects of amyloid deposits.

The mechanism by which β_2_m fibrils kill cells is unknown, although other amyloid fibrils have been reported to cause both necrotic and apoptopic cell death [44]–[46]. β_2_m fibrils disrupt purified lipid membranes [29] suggesting a role for membrane disruption in fibril-associated cytotoxicity. Since β_2_m fibrils were visualized in association with monocytes, osteoblasts and chondrocytes in proximity to the plasma membrane and within intracellular punctate structures, β_2_m fibrils could kill these cells by disrupting the plasma membrane and/or intracellular membranes. No immunoreactivity was detected by the antibody A11, which recognises amyloid oligomers [32]. This suggests that cytotoxicity associated with β_2_m fibril preparation is mediated by the interaction of β_2_m fibrils with the cells.

Incubation of cells with monomeric WT β_2_m or ΔN6β_2_m also resulted in reduced viability, with the ΔN6β_2_m monomer exhibiting the greatest degree of cytotoxicity. While, no oligomers were detected in either protein preparation as determined by SEC and by immunoblotting with the oligomer specific antibody A11, WT β_2_m can form cytotoxic non-fibrillar aggregates [47], [48], whereas the cytotoxicity of ΔN6β_2_m could reflect the increased propensity of this truncation to aggregate or its different conformational properties [12], [13], [15]–[18]. Although elevated concentrations of WT β_2_m are characteristic of DRA [2], [3], [7], the relevance of non-fibrillar ΔN6β_2_m cytotoxicity is unclear, as ΔN6β_2_m has only been detected in amyloid deposits and is not found in the serum of hemodialysis patients [15], [49]. ΔN6β_2_m, however, has an increased affinity for collagen [50], which could lead to localized high concentrations of the soluble protein and resultant cytotoxicity.

The study outlined herein examined how monomeric and fibrillar β_2_m affect primary human cells present in the osteoarticular tissues of subjects with DRA. We have shown previously that β_2_m fibrils are cytotoxic to the murine macrophage RAW 264.7 and human neuroblastoma SH-SY5Y cell lines [29], demonstrating that β_2_m fibril-associated cytotoxicity is not unique to monocytes, osteoblasts and chondrocytes. Furthermore, since fibrillar preparations of other amyloidogenic precursors are also cytotoxic [29], [44]–[46], [51]–[54], it is likely that these would reduce viability of monocytes, osteoblasts and chondrocytes. Amyloid deposits in the osteoarticular tissues can be present in other amyloid diseases [55], [56] and may cause the death of osteoblasts and chondrocytes. Macrophages and macrophage-like cells such as microglia are found in association with other forms of amyloid [57], [58]. As such fibril-associated cytotoxicity to macrophages and macrophage-like cells could be a factor in other amyloid diseases.

In conclusion, our observations show that cytotoxicity of monomeric β_2_m and β_2_m fibrils to cell types in the joints of subjects with DRA could be important factors in the development of the osteoarticular pathology of this disorder (Fig. 6).

## Materials and Methods

### Ethics statement

The procedure for the isolation of monocytes from peripheral blood of healthy volunteers was approved by the Faculty of Biological Sciences Research Ethics Committee at the University of Leeds. Written informed consent was given by each donor.

### Expression, purification and labelling of β_2_m

Recombinant WT β_2_m and ΔN6β_2_m were expressed in *E. coli* and purified as described previously [12], [30], [31]. Purification was confirmed by electrospray ionization mass spectrometry and size exclusion chromatography, as previously described [17]. Endotoxins were removed using an “EndoTrap Red” column (Hyglos, Bernreid, Germany) and Limulus amoebocyte lysate assays performed by Lonza (Verviers, Belgium) showed endotoxin concentrations to be ≤7.35 EU/mg protein. Purified proteins were labelled with TMR (Molecular Probes, Eugene, OR, USA) using methods described previously [40]. Further details are provided in File S1.

### Generation of β_2_m fibrils at physiological pH

Fibrils formed at pH 2 [29] were fragmented by freezing in liquid nitrogen and thawing at 37°C three times to generate fibril seeds. 5% (v/v) seeds were elongated with 1 mg/ml ΔN6β_2_m in Dulbecco's Phosphate Buffered Saline (PBS) pH 7.3, at 37°C, with agitation at 200 rpm in the presence of 0.1 mg/ml low molecular weight heparin (Sigma-Aldrich H8537), 100 U/ml penicillin and 100 µg/ml streptomycin, for 21 days. To generate labelled fibrils, seeds were extended with 0.9 mg/ml unlabelled ΔN6β_2_m and 0.1 mg/ml TMR-ΔN6β_2_m. Fibril concentrations are stated as monomer equivalents.

### TEM, thioflavin-T binding and immunoblotting

Negative stain TEM and thioflavin-T assays were performed as previously described [17], [30]. Immunoblotting with A11 and WO1 antibodies was performed as described previously [32], [33], [59], and in detail in File S1.

### Isolation and culture of primary human monocytes

Mononuclear cells were isolated from peripheral blood by centrifugation over Lymphoprep (Axis-Shield, Oslo, Norway), monocytes were enriched using the MACS monocyte II kit (Miltenyi Biotec, Bergisch Gladbach, Germany) and enrichment confirmed by flow cytometric analysis of cells stained with anti-CD14-Phycoerythrin (Miltenyi Biotec) on a FACSCalibur instrument (BD Biosciences). Monocytes were cultured at 500 000 cells/cm^2^, in αMEM supplemented with 10% (v/v) fetal bovine serum (Biosera, Ringmer, UK), 2 mM L-glutamine, 100 U/ml penicillin, 100 µg/ml streptomycin and 750 ng/ml M-CSF (Peprotech, Rocky Hill, NJ, USA), at 37°C, 5% CO_2_.

### Culture of primary human chondrocytes and osteoblasts

Primary human osteoblasts, chondrocytes, cell specific media and an optimized cell detach kit were obtained from PromoCell (Heidelberg, Germany). Cells were cultured at 10 000–20 000 cells/cm^2^, in cell specific media supplemented with 100 U/ml penicillin and 100 µg/ml streptomycin at 37°C, 5% CO_2_. Cells were passaged a maximum of three times.

### MTT assay of cell viability

Cells were plated in 96 well plates (Corning Inc., Corning, NY, USA) and cultured overnight. MTT assays were then performed as described previously [29] and in File S1. For each cell type MTT assays were performed using the same culture conditions, enabling each experiment to be internally controlled to exclude any effects of the growth medium on the reduction of MTT.

### Imaging of β_2_m internalization and degradation

Uptake of 10 µg/ml ΔN6β_2_m monomer (90% unlabelled, 10% TMR labelled) or TMR-ΔN6β_2_m fibrils over 24 hours, and degradation over a further 24 hours was analyzed using methods described previously [40] and in detail in File S1.

### Osteoclast formation and characterization

Primary human monocytes were cultured in 96 well plates (Corning) or on 16 well osteologic slides (BD Biosciences). Osteoclast formation was assessed after 14 days by staining for TRAP and counterstaining with hematoxylin, using a leukocyte acid phosphatase (TRAP) kit (Sigma-Aldrich 387A) and manufacturer's protocol, and by quantifying the percentage resorption of the osteologic substrate. Further details are provided in File S1.

## Supporting Information

Figure S1 **Effects of monomeric and fibrillar β_2_-microglobulin (β_2_m) on monocyte viability.** Primary human monocytes from three independent donors were treated with the indicated substances for 24 hours and then their ability to reduce MTT, as a measure of viability, was measured for each donor. (a) Effects of ΔN6β_2_m (ΔN6) monomer and β_2_m fibrils on viability. (b) Effect of full-length wild type (WT) β_2_m monomer on viability. Error bars represent the SEM; P values differing from the PBS control were calculated by the Student's t-test, * = P≤0.01; ** = P≤0.001.
(TIFF)

Figure S2 **Monomeric and fibrillar β_2_-microglobulin (β_2_m) association with osteoblasts and their effects on cell viability.** (a) Primary human osteoblasts were incubated with either TMR-labelled ΔN6β_2_m monomer or TMR-labelled β_2_m fibrils (red) for 24 hours and then imaged by confocal microscopy. Differential interferance contrast (DIC) images are also shown. The regions within the white boxes are magnified in the right hand panels. (b) Cells from four independent donors were treated with ΔN6β_2_m monomer or β_2_m fibrils for 24 hours and then their ability to reduce MTT, as a measure of viability, was measured for each donor. Error bars represent the SEM; P values differing from the PBS control were calculated by the Student's t-test, * = P≤0.01; ** = P≤0.001.
(TIFF)

Figure S3 **Monomeric and fibrillar β_2_-microglobulin (β_2_m) association with chondrocytes and their effects on cell viability.** (a) Primary human chondrocytes were treated with either TMR-labelled ΔN6β_2_m monomer or TMR-labelled fibrils (red) for 24 hours and then imaged by confocal microscopy. Differential interferance contrast (DIC) images are also shown. The regions within the white boxes are magnified in the in the right hand panels. (b) Chondrocytes from four independent donors were treated with ΔN6β_2_m monomer or β_2_m fibrils for 24 hours and then their ability to reduce MTT, as a measure of viability, was measured for each donor. Error bars represent the SEM; P values differing from the PBS control were calculated by the Student's t-test, * = P≤0.01; ** = P≤0.001.
(TIFF)

File S1 Full materials and methods.
(DOC)
