# Supplementary figures and images for: Characterization of the Response of Primary Cells Relevant to Dialysis-Related Amyloidosis to β2-Microglobulin Monomer and Fibrils
Source: PLoS One. 2011 Nov 9;6(11):e27353. doi: 10.1371/journal.pone.0027353 (PMC3212568; doi:10.1371/journal.pone.0027353)

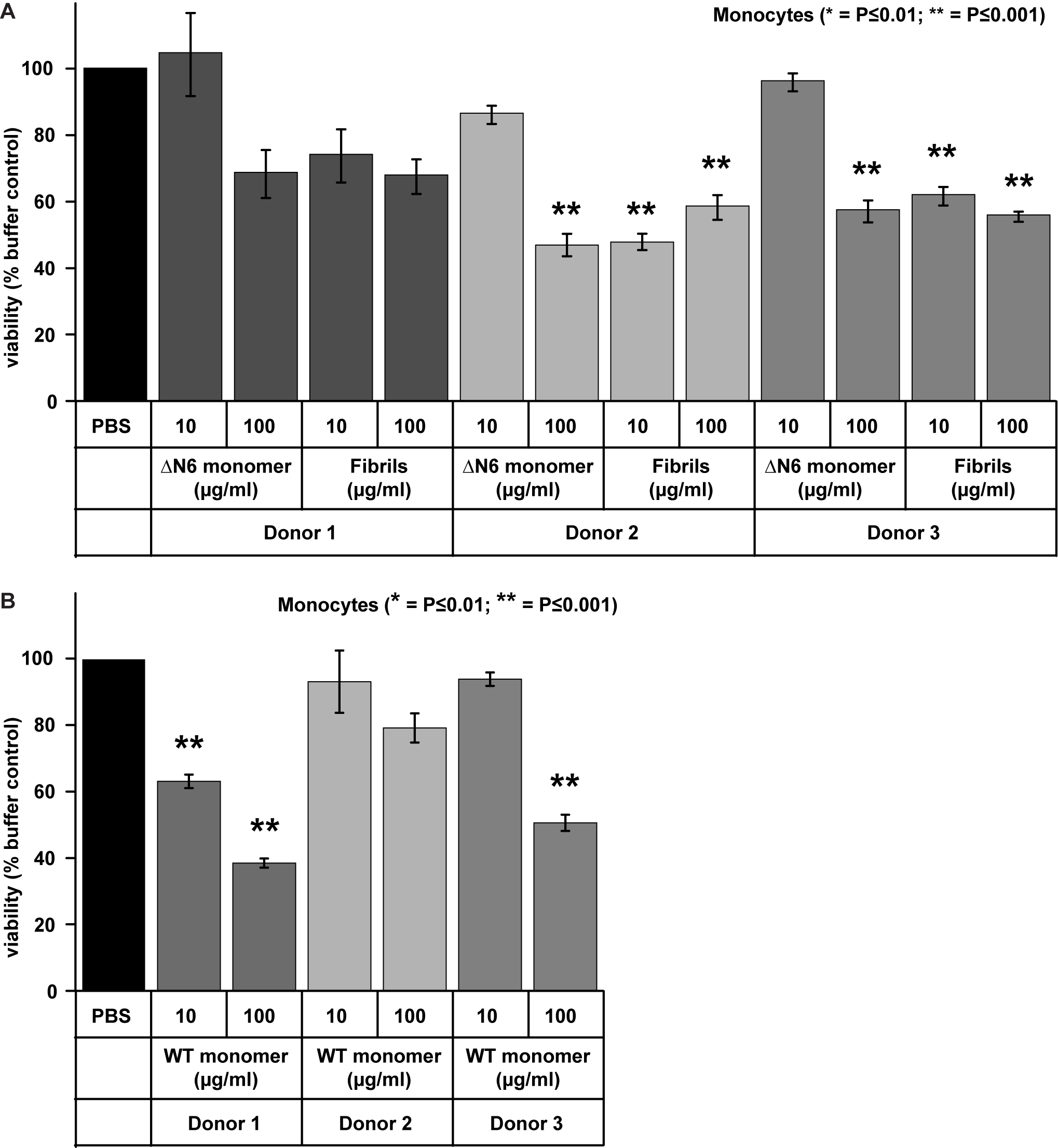

Supplement: Figure S1 — Effects of monomeric and fibrillar β2-microglobulin (β2m) on monocyte viability. Primary human monocytes from three independent donors were treated with the indicated substances for 24 hours and then their ability to reduce MTT, as a measure of viability, was measured for each donor. (a) Effects of ΔN6β2m (ΔN6) monomer and β2m fibrils on viability. (b) Effect of full-length wild type (WT) β2m monomer on viability. Error bars represent the SEM; P values differing from the PBS control were calculated by the Student's t-test, * = P≤0.01; ** = P≤0.001. (TIFF) [file pone.0027353.s001.tiff]

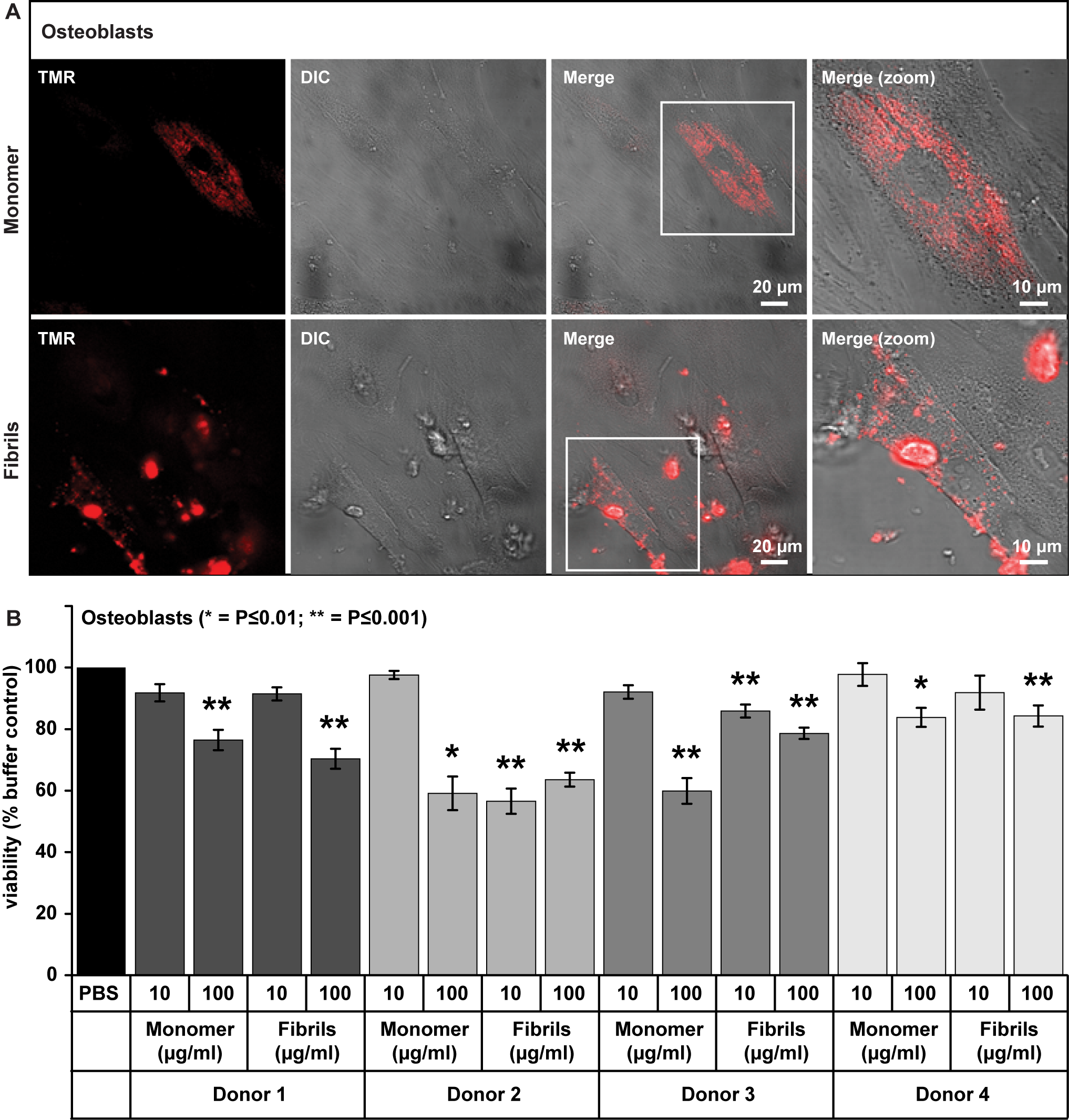

Supplement: Figure S2 — Monomeric and fibrillar β2-microglobulin (β2m) association with osteoblasts and their effects on cell viability. (a) Primary human osteoblasts were incubated with either TMR-labelled ΔN6β2m monomer or TMR-labelled β2m fibrils (red) for 24 hours and then imaged by confocal microscopy. Differential interferance contrast (DIC) images are also shown. The regions within the white boxes are magnified in the right hand panels. (b) Cells from four independent donors were treated with ΔN6β2m monomer or β2m fibrils for 24 hours and then their ability to reduce MTT, as a measure of viability, was measured for each donor. Error bars represent the SEM; P values differing from the PBS control were calculated by the Student's t-test, * = P≤0.01; ** = P≤0.001. (TIFF) [file pone.0027353.s002.tiff]

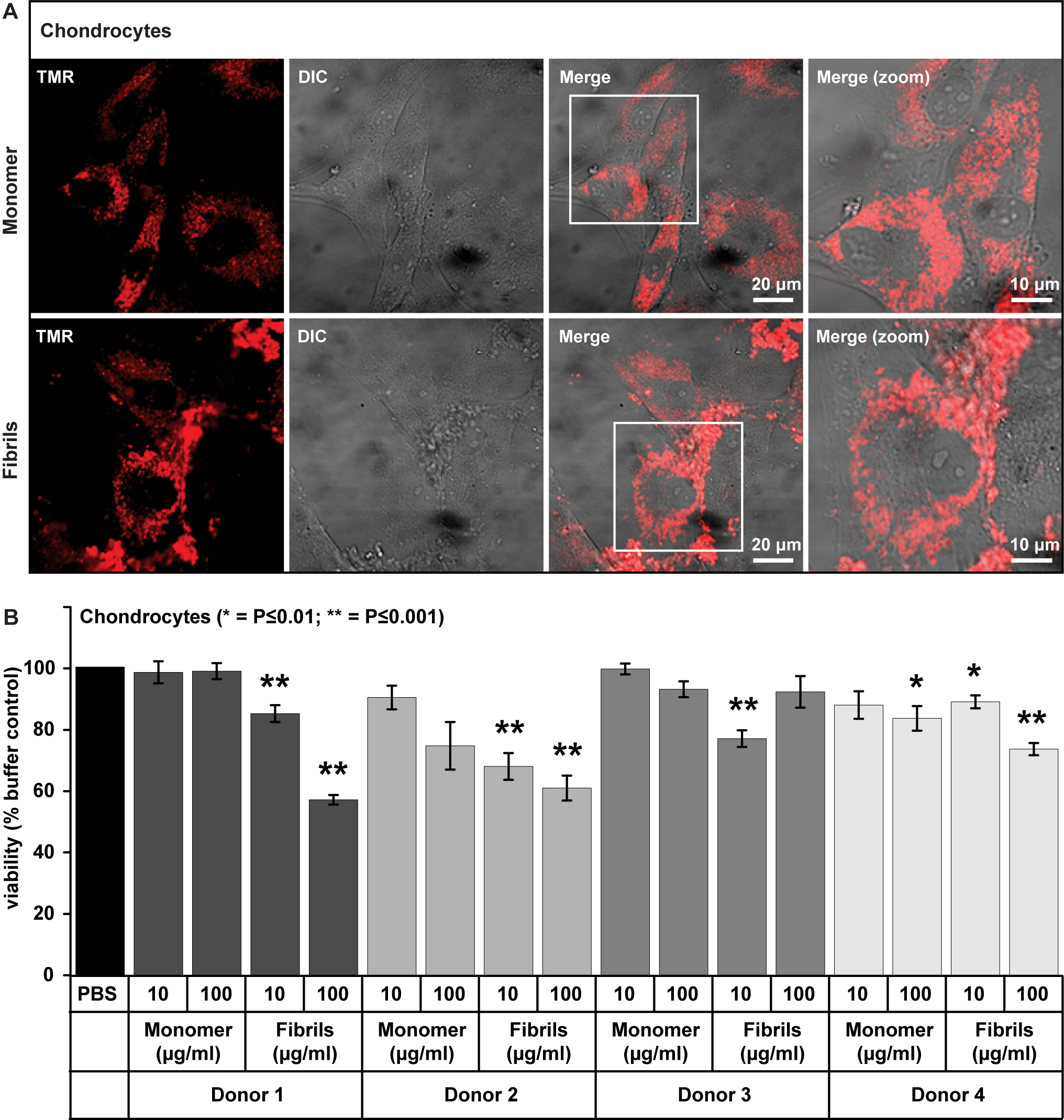

Supplement: Figure S3 — Monomeric and fibrillar β2-microglobulin (β2m) association with chondrocytes and their effects on cell viability. (a) Primary human chondrocytes were treated with either TMR-labelled ΔN6β2m monomer or TMR-labelled fibrils (red) for 24 hours and then imaged by confocal microscopy. Differential interferance contrast (DIC) images are also shown. The regions within the white boxes are magnified in the in the right hand panels. (b) Chondrocytes from four independent donors were treated with ΔN6β2m monomer or β2m fibrils for 24 hours and then their ability to reduce MTT, as a measure of viability, was measured for each donor. Error bars represent the SEM; P values differing from the PBS control were calculated by the Student's t-test, * = P≤0.01; ** = P≤0.001. (TIFF) [file pone.0027353.s003.tiff]
