# Supplementary material for: Characterization of the Response of Primary Cells Relevant to Dialysis-Related Amyloidosis to β2-Microglobulin Monomer and Fibrils
Source: PLoS One. 2011 Nov 9;6(11):e27353. doi: 10.1371/journal.pone.0027353 (PMC3212568; doi:10.1371/journal.pone.0027353)
Supplement: File S1 — Full materials and methods. (DOC) [file pone.0027353.s004.doc]

**File S1**

**Characterization of the response of primary cells relevant to dialysis-related amyloidosis to β_2_-microglobulin monomer and fibrils**

Morwenna Y. Porter, Katy E. Routledge, Sheena E. Radford and Eric W. Hewitt

**Full Materials and methods**

***Materials***

Unless otherwise stated, all reagents were purchased from Sigma Aldrich (Poole, Dorset UK).

### *Expression and purification of β_2_-microglobulin (β_2_m)*

### Recombinant wild type (WT) β_2_m and ΔN6β_2_m were expressed in the *E. coli* strain BL21(DE3)pLysS from plasmids pINK(WT) and pINK(ΔN6) respectively. Cells were lyzed in lysis buffer (25 mM Tris-HCl, pH 8, 100 µg/ml lysozyme, 50 µg/ml PMSF, 20 µg/ml DNase, 1mM EDTA) by passage through a cell disrupter (Constant Systems, Daventry, UK) and inclusion bodies isolated by centrifugation at 23 000 g for 30 minutes at 4°C. Inclusion bodies were washed four times in 25mM Tris HCl, pH 8.0, the expressed protein solubilised in 8M urea in 25mM Tris HCl, pH 8 and then refolded by dialysis against three changes of 5 l 25 mM Tris-HCl at pH 8 for WT β_2_m, pH 9.0 for ΔN6β_2_m. The refolded protein was purified by ion exchange chromatography, using a Q-Sepharose column (GE Healthcare Life Sciences, Little Chalfont, UK) and an AKTA prime plus pump system (GE Healthcare): Following equilibration in buffer (25 mM Tris-HCl, at pH 8 for WT β_2_m, pH 9.0 for ΔN6β_2_m), the protein was loaded, the column washed with the same buffer and the protein eluted over a one litre 0- 400 mM NaCl gradient in 25 mM Tris-HCl at pH8.0 for both WT β_2_m and ΔN6β_2_m. Fractions containing the purified WT β_2_m or ΔN6β_2_m protein were pooled, dialyzed against three changes of 5 l deionized water and lyophilized. Lyophilized protein was resuspended in 25 mM sodium phosphate, pH 7.5 to a final concentration of 10 mg/ml (900 µM) and further purified by gel filtration using a Superdex75 HiLoad column attached to an AKTA prime plus pump system (GE Healthcare): After equilibration in 25 mM sodium phosphate, pH 7.5, the protein was applied via injection into a 5 ml external loop at 3 ml/min. Fractions containing the purified protein were pooled and WT β_2_m protein was lyophilized and stored at -20°C, while ΔN6β_2_m was concentrated to 7.5 mg/ml (630 µM) using Centriprep concentrators (Millipore, Billerica, MA, USA) and stored in solution at -80°C.

### To confirm protein identity, 10 µg of protein was buffer exchanged into 80% acetonitrile/0.1% formic acid using C18 resin ZipTips (Millipore) and analyzed by electrospray ionization mass spectrometry (ESI-MS) on a Synapt HDMS mass spectrometer (Waters, Elstree, UK).

### To remove endotoxin, proteins in sterile Dulbecco’s phosphate buffered saline (PBS) (WT β_2_m) or 25 mM sodium phosphate, pH 7.5 (ΔN6β_2_m) were applied to an EndoTrap Red column (Hyglos, Bernreid, Germany), following the manufacturer’s protocol, eluted in Dulbecco’s PBS and stored at -80°C. Limulus amoebocyte lysate assays were performed by Lonza (Verviers, Belgium); endotoxin concentrations in all preparations were ≤ 7.35 EU/mg protein.

***Analytical Size Exclusion Chromatography (SEC)***

SEC was performed using a Superdex75 10/300 GL column attached to an AKTA prime plus pump system (GE Healthcare). The column was washed in two column volumes of filtered and degassed deionized water, equilibrated with two column volumes of filtered and degassed PBS, pH 7.3 (Oxoid, Cambridge, UK) and then 100 µl of 1 mg/ml protein filtered through a 0.2 µm inorganic membrane, (Whatman, Maidstone, UK) was applied to the column at a rate of 0.4 ml/min at room temperature. Monomeric WT β_2_m eluted at a retention volume of ~ 14.6 ml, while ΔN6β_2_m eluted at ~ 15 ml.

***Labelling β_2_m with 5-(and -6)-carboxytetramethylrhodamine-SE (TMR)***

A ten-fold molar excess of TMR (Molecular Probes, Eugene, OR, USA) in dimethylsulfoxide was added to 2.4 mg/ml ΔN6β_2_m in PBS, and incubated, stirring at room temperature for 45 min. Excess dye was removed with a PD10 desalting column (GE Healthcare Life Sciences). The column was equilibrated with Dulbecco’s PBS, the protein applied and then eluted in the same buffer. Aliquots were stored at -80**°**C. Incorporation of 1, 2, 3, 4 and 5 molecules of TMR, at a ratio of ~1:2:3:2:1 was confirmed by ESI-MS.

***Generation of β_2_m fibrils under physiological conditions***

To generate WT β_2_m fibrils, the pH of the sterile protein at 1.3 mg/ml (110 µM) in Dulbecco’s PBS was dropped to pH 2.0 using orthophosphoric acid and this incubated in the presence of 100 U/ml penicillin and 100 µg/ml streptomycin at 37°C, with agitation at 200 rpm, for 7 days. Fibrils formed were fragmented by freezing in liquid nitrogen and thawing at 37°C three times to generate seeds. 5% (v/v) seeds were then elongated with 1 mg/ml ΔN6β_2_m in Dulbecco’s PBS at pH 7.3, 37°C, with agitation at 200 rpm in the presence of 0.1 mg/ml low molecular weight heparin (Sigma-Aldrich H8537), 100 U/ml penicillin and 100 µg/ml streptomycin, for 21 days. To generate labelled fibrils, seeds were extended with 0.9 mg/ml unlabelled ΔN6β_2_m and 0.1 mg/ml TMR-ΔN6β_2_m. Fibril concentrations are stated as monomer equivalents.

***Transmission electron microscopy***

Carbon coated copper grids (Agar Scientific, Stanstead, UK) were placed on 10 µl drops of each sample for 30 s, excess liquid removed by blotting with filter paper and the samples stained for 45 s on a drop of 4% uranyl acetate. Grids were blotted, air-dried and analyzed on a Philips CM10 electron microscope operating at 80 keV.

***Thioflavin-T binding assays***

10 µl each sample was added to 1 ml thioflavin-T buffer (10 µM ThT, 0.5M Tris-HCl, pH 8.5), and binding quantified by measuring fluorescence emission at 480 nm, following excitation at 444 on a QM-1 spectrofluorimmeter (Photon Technology International, Ford, UK). 60 readings were taken over 1 minute and the average +/- 1SD was plotted.

***Immunoblotting***

2 μg each sample was spotted onto nitrocellulose membrane (GE Healthcare) and dried at room temperature for 1 hour. Membranes were blocked in 10% (w/v) dried milk powder in buffer (A11 buffer: PBS + 0.01% (v/v) Tween-20; WO1 buffer: PBS + (v/v) 0.2% Tween-20) for 1 hour at room temperature (WO1) or overnight at 4˚C (A11), washed three times in buffer and then incubated in either 1:5000 A11 antibody (a kind gift from C. Glabe, University of California, Irvine) in buffer plus 5% (w/v) dried milk powder for 1 hour at room temperature or 1:5000 WO1 antibody (a kind gift from R. Wetzel, University of Pittsburgh) in buffer plus 3% (w/v) bovine serum albumin overnight at 4˚C. Membranes were then washed three times in buffer, incubated in secondary antibody (1:5000 horseradish peroxidise (HRP) conjugated goat anti-rabbit (BioRad, Hemel Hempstead, UK) for A11, 1:5000 HRP conjugated rat anti-mouse (BD Biosciences, Franklin Lakes, NJ, USA) for WO1) for 1 hour at room temperature, washed three times in buffer and developed using SuperSignal West Pico Chemiluminescent substrate (Thermo Scientific, Rockford, IL, USA) and x-ray film (SLS, Nottingham, UK). Aβ_1-40_ oligomers (C. Glabe) were used as a positive control for A11.

***Isolation and culture of primary human monocytes***

Fresh peripheral blood was obtained from healthy volunteers. Ethical permission for this procedure was provided by the Faculty of Biological Sciences ethics committee, University of Leeds. Mononuclear cells were isolated by centrifugation over Lymphoprep (Axis-Shield, Oslo, Norway), and monocytes were then enriched with the MACS monocyte II kit (Miltenyi Biotec, Bergisch Gladbach, Germany). CD14^+^ cell enrichment was confirmed by flow cytometric analysis of cells stained with anti-CD14-Phycoerythrin (Miltenyi Biotec) on a FACSCalibur instrument (BD Biosciences). Monocytes were cultured at a density of 500 000 cells/cm^2^, in αMEM supplemented with 10% (v/v) fetal bovine serum (Biosera, Ringmer, UK), 2 mM L-glutamine, 100 U/ml penicillin, 100 µg/ml streptomycin and 750 ng/ml mCSF (Peprotech, Rocky Hill, NJ, USA), at 37°C, 5% CO_2_.

***Culture of primary human chondrocytes and osteoblasts***

Primary human osteoblasts, chondrocytes, cell specific media and an optimized cell detach kit were obtained from PromoCell (Heidelberg, Germany). Cells were cultured according to the company’s protocols at a density of 10 000-20 000 cells/cm^2^, in cell specific media supplemented with 100 U/ml penicillin and 100 µg/ml streptomycin at 37°C, 5% CO_2_. Cells were passaged a maximum of three times.

***3-(4,5-dimethyl-2-thiazolyl)-2,5-diphenyl-2H-tetrazolium bromide (MTT) assay of cell viability***

Cells were plated in 96 well plates (Corning Inc., Corning, NY, USA), cultured overnight and then incubated with control and test substances for 24 hours. MTT was then added to the cell culture media at a final concentration of 0.8 mg/ml, incubated with cells for approximately three hours until formazan crystals were formed and these crystals dissolved in 50 µl DMSO. The absorption at 570 nm was measured and the background signal, measured at 650 nm, subtracted using a Powerwave XS2 plate reader (BioTek, Potton, UK) with Gen5 software (BioTek). Results were normalized by setting the viability of cells treated with 10 µl PBS alone as 100% viability and cells treated with 10 µl 20% (w/v) sodium azide as 0% viability. A minimum of four replicates were performed per condition. The mean and standard error for each condition was calculated. Statistical significance was calculated using the Student’s T-test. Heparin, which was used to stabilise fibril seeds, was present in the β_2_m fibril preparations incubated with cells. The resultant concentration of heparin added to the cell culture medium with 10 or 100 mg/ml fibrils was 14 ng/ml or 140 ng/ml respectively. In controls, neither concentration of heparin when added with PBS had any effect on the viability of any cell type analysed in this study.

***Imaging of β_2_m internalization and degradation***

Cells were cultured overnight in 96 well µicroclear plates (Greiner Bio-one, Frickenhausen, Germany) before 10 µg/ml ΔN6β_2_m monomer (90% unlabelled ΔN6β_2_m, 10% TMR labelled ΔN6β_2_m) or TMR-ΔN6β_2_m fibrils were added. Cells were incubated for a further 24 hours to allow uptake of labelled proteins and then imaged on a LSM510 Meta confocal microscope (Zeiss, Welwyn Garden City, UK). Before imaging, cells were incubated with 50 nM of LysoTracker Green (Molecular Probes, Eugene, OR,USA) for 1 hour. To determine the extent of monomer and fibril degradation, cells incubated with labelled protein for 24 hours were washed twice in media, incubated in the absence of labelled protein for a further 24 hours and imaged as above. Percentage uptake and degradation was determined using ImageJ software (NIH, Bethesda, MD, USA). Cells were delineated by hand and internal TMR fluorescence measured.

***Osteoclast formation and characterization***

Primary human monocytes were isolated and cultured in 96 well plates (Corning, Amsterdam, Netherlands) or on 16 well osteologic slides (BD Biosciences). After 24 hours, media was replaced and control or test samples added. Media was replaced every 2-3 days and fresh control and test samples were added each time. The negative control was 10 µl PBS, the positive control was 375 ng/ml RANKL (Peprotech). After 14 days the formation of large, multinucleate, TRAP positive osteoclasts, which could resorb the osteologic substrate was assessed. Cells in 96 well plates were fixed in 3% (v/v) formaldehyde, stained for TRAP and counter stained with haematoxylin using a leukocyte acid phosphatase (TRAP) kit (Sigma-Aldrich 387A) and manufacturer’s protocol. Cells were imaged on a DM IL inverted microscope (Leica Microsystems, Milton Keynes, UK). Cells on osteologic slides were removed using a 5% (v/v) bleach solution. The slides were then washed in distilled water, imaged on a LSM 510 Meta confocal (Zeiss), and the percent resorption of the osteologic surface calculated using Image J software (NIH).

1. Kad NM, Thomson NH, Smith DP, Smith DA, Radford SE (2001) β_2_-microglobulin and its deamidated variant, N17D form amyloid fibrils with a range of morphologies in vitro. J Mol Biol 313: 559-571.

2. Myers SL, Jones S, Jahn TR, Morten IJ, Tennent GA, et al. (2006) A systematic study of the effect of physiological factors on β_2_-microglobulin amyloid formation at neutral pH. Biochemistry 45: 2311-2321.

3. Kayed R, Head E, Thompson JL, McIntire TM, Milton SC, et al. (2003) Common structure of soluble amyloid oligomers implies common mechanism of pathogenesis. Science 300: 486-489.

4. O'Nuallain B, Wetzel R (2002) Conformational Abs recognizing a generic amyloid fibril epitope. Proc Natl Acad Sci U S A 99: 1485-1490.
